# Stroke caused by cerebral amyloid angiopathy: a case of difficult diagnosis

**DOI:** 10.1590/1980-5764-DN-2025-0288

**Published:** 2025-12-19

**Authors:** Rocío Campos Gamarra, Carla Villanueva Colina, Jimmy Palacios-García

**Affiliations:** 1 Universidad Peruana Cayetano Heredia, Lima, Peru.; 2 Clínica Angloamericana, Lima, Peru.

**Keywords:** Cerebral Amyloid Angiopathy, Cerebral Hemorrhage, Stroke, Ischemic Stroke, Angiopatía Amiloide Cerebral, Hemorragia Cerebral, Accidente Cerebrovascular, Accidente Cerebrovascular Isquémico

## Abstract

Cerebral amyloid angiopathy is a common cause of intracerebral hemorrhage, most often occurring in older adults and in patients with dementia. One of its main differential diagnoses is hypertensive intracerebral hemorrhage. This is a case report of a hypertensive male patient who presented to the emergency department with short-term amnesia and Broca’s aphasia. The initial diagnosis was hemorrhagic stroke of hypertensive etiology. However, subsequent diagnostic tests revealed that the bleeding was due to amyloid angiopathy. This case highlights the similarities and differences in the diagnosis and management of both etiologies, emphasizing the importance of establishing an accurate and timely differential diagnosis.

## RESUMEN.

La angiopatía amiloide cerebral es una causa frecuente de hemorragia intracerebral, que ocurre con mayor frecuencia en adultos mayores y en pacientes con demencia. Uno de sus principales diagnósticos diferenciales es la hemorragia intracerebral de origen hipertensivo. Este informe describió el caso de un paciente masculino hipertenso que acudió al servicio de urgencias con amnesia de corta duración y afasia de Broca. El diagnóstico inicial fue de accidente cerebrovascular hemorrágico de etiología hipertensiva. Sin embargo, pruebas diagnósticas posteriores revelaron que el sangrado se debía a una angiopatía amiloide. Este informe sirve para destacar las similitudes y diferencias en el diagnóstico y manejo de ambas etiologías, enfatizando la importancia de establecer un diagnóstico diferencial preciso y oportuno.

## INTRODUCTION

 Cerebral amyloid angiopathy (CAA) is a cerebrovascular disorder characterized by deposits of beta-amyloid peptide in small- to medium-caliber vessels of the brain and leptomeninges. 

 CAA may present with clinical features that mimic other stroke subtypes. Therefore, evaluation of its epidemiological and radiological characteristics is necessary to enable differential diagnosis and facilitate timely intervention, thereby improving patient prognosis. CAA carries a higher risk of recurrent bleeding than that associated with arterial hypertension^1^. 

 This report aimed to describe the clinical and radiological characteristics of a case of CAA in which the main differential diagnosis was hemorrhagic stroke. This article was prepared in accordance with the CARE guidelines^2^. 

## CASE REPORT

 A 78-year-old Latino male businessman with a medical history of arterial hypertension and glaucoma presented to the Emergency Department of a private clinic with a 10-hour history of illness characterized by short-term amnesia and Broca’s aphasia. He was receiving regular treatment with valsartan 80 mg, acetylsalicylic acid 100 mg, and rosuvastatin 10 mg every 24 hours. He denied any family or psychosocial history, as well as previous surgeries. On physical examination, the patient was hemodynamically stable. Neurological examination revealed mild difficulty in object naming with paraphasias and short-term memory impairment. No motor or sensory deficits were observed. The patient received intravenous hydration, omeprazole, valsartan, and labetalol. Aspirin and rosuvastatin were discontinued. A brain computed tomography (CT) angiography was performed upon admission, revealing an intraparenchymal hematoma in the left temporal lobe, associated with surrounding vasogenic edema, without midline deviation. A cervical CT angiography was also performed, showing no abnormalities. Acetylcysteine was prescribed for neuroprotection due to contrast use. 

**Figure 1:**
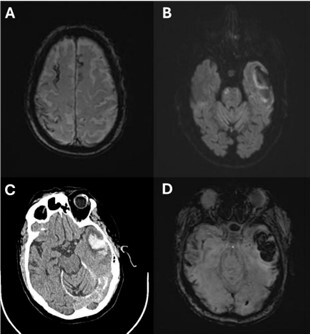
The brain MRI revealed a hypointense image suggestive of superficial siderosis. The brain CT sequence revealed a hyperdense image suggestive of bleeding.

 The following day, a contrast-enhanced brain magnetic resonance imaging (MRI) was performed (Figure 1A) due to the suspicion of a bleeding tumor lesion. The MRI revealed a hemorrhagic lesion, described by radiology as having a central necrotic and hematologic appearance, with signs of perilesional edema at the left temporal level. It also showed increased intralesional perfusion and nonspecific changes in the metabolite peak ratios on spectroscopy — findings suggestive of trophic changes, leukoaraiosis with a sequelae-like appearance, and signs of a right frontal venous angioma. A routine electroencephalogram showed no abnormalities. 

(A) MRI of the brain with contrast with magnetic susceptibility cortical view shows signs of bilateral frontal and cortical siderosis with predominance on the right hemisphere. (B) MRI of the brain with contrast in diffusion sequence (DWI) shows restriction to diffusion in the right cerebral pons region, and signs of hemorrhagic injury with a necrotic hematical appearance center with signs of perilesional edema at the left temporal level. (C) CT scan shows subacute intraparenchymal hemorrhage in the left temporal region with decreased vasogenic edema. (D) Magnetic susceptibility weighted image (SWI) showing lobar bleeding in the left temporal region, in addition to a small old left occipital bleeding.

 On day 3 of hospitalization, a 3D angiography of the brain and supra-aortic trunks was performed. No evidence of vascular injury associated with the cerebral bleeding was identified. However, stenosis greater than 90% at the origin of the right vertebral artery was observed. The study also reported 50% stenosis at the origin of the right internal carotid artery at the carotid bulb and 25% stenosis at the C2 cervical segment, along with signs of intra- and extracranial atherosclerosis. A few hours after the procedure, the patient experienced nausea and one episode of vomiting, attributed to contrast agent use; acetylcysteine and granisetron were administered. Later that night, the patient developed delirium, likely due to residual effects of medication, for which haloperidol was given. 

 One day after the procedure, the patient developed new-onset dysarthria, left facial paresis, and somnolence. Motor strength evaluation revealed decreased strength in the upper limbs (⅘) and lower limbs (⅗) bilaterally. The Glasgow Coma Scale (GCS) score was 9/15. Due to the appearance of new neurological symptoms, a follow-up MRI was performed (Figure 1B). The MRI revealed acute signs of multiple punctate ischemic lesions in both cerebral hemispheres, both cerebellar hemispheres, and the pons. Additionally, signal alterations suggestive of sequelae-like appearance were observed in the sulci of both frontal and the right parietal lobes. At the temporal level, there were signs of a hemorrhagic lesion with peripheral areas of restricted diffusion, indicating hypoxic injury of undetermined etiology, accompanied by perilesional edema. The following day, a further decline in neurological status was evident. The patient presented with decreased consciousness, mobilized his legs only in response to tactile stimulation, and continued to have dysarthria. No meningeal signs were found. It was considered that the new neurological symptoms were a complication related to the angiographic procedure. A nasogastric tube was placed, mannitol therapy was initiated for vasogenic edema, and neurological monitoring was implemented. 

 A brain CT was performed (Figure 1C), showing persistent subacute hemorrhagic involvement in the left temporal lobe, increased surrounding vasogenic edema, and a decrease in subarachnoid hemorrhage in the same region. It also demonstrated resolution of multifocal cortical hyperintensities with a gyriform pattern in the left frontal lobe. A transcranial Doppler ultrasound revealed cerebral arterial flow within normal limits, suggestive of microangiopathy. An echocardiogram showed a left ventricular ejection fraction (EF) of 70%, borderline myocardial thickness, aortic valve calcification, and no evidence of thrombi or vegetations. A doppler ultrasound of the carotid and supra-aortic trunks was performed to rule out the possibility of carotid plaque dislodgement during angiography and to assess arterial flow in the carotid-vertebral-cerebral arteries. The exam revealed atheromatosis of the bilateral common carotid arteries and the right internal carotid artery, with approximately 40% stenosis in the proximal segment of the right internal carotid artery. The right vertebral artery was patent but smaller in caliber and showed a reduced spectral waveform compared to the contralateral side. The common carotid and right external carotid arteries were patent, and the extracranial carotid and left vertebral-subclavian arterial axes showed good patency. 

 After two days, the patient showed clinical improvement. He was awake, followed commands, and cooperated during the physical examination; however, dysarthria and intermittent somnolence persisted. A new CT scan revealed persistent subacute hemorrhagic involvement in the left temporal lobe and resorption of the subarachnoid hemorrhage at the same location. An arteriovenous ultrasound of the lower limbs revealed no significant findings. The patient’s neurological status progressed slowly but favorably. He remained oriented in space, time, and partially in person, and the somnolence resolved. A follow-up non-contrast CT scan revealed a subacute intraparenchymal hemorrhage in the left temporal region with decreased vasogenic edema compared to the previous study. The patient achieved a GCS score of 15/15 and was subsequently discharged. 

 The MRI showed evidence of old cortical bleeding (Figure 1D), indicating that the patient meets the Boston Criteria 2.0 for the diagnosis of CAA. 

## DISCUSSION

 CAA is a progressive disease that primarily affects aged patients, leading to intracerebral hemorrhage (ICH) and cognitive impairment. It is characterized by amyloid-β deposition in small- and medium-sized cortical and leptomeningeal vessels. The prevalence of CAA increases with age^3^ and is higher in patients with a concomitant diagnosis of dementia^4^ or Alzheimer disease^5^. 

 CAA is strongly associated with ICH, its most significant clinical manifestation^6^, and accounts for 5–20% of non-traumatic ICH in the elderly. CAA-related hemorrhages are typically lobar and may be multiple or recurrent^7^. In vitro studies have shown that CAA may increase the risk of ICH following thrombolytic therapy, likely due to amyloid-related degeneration of blood vessel walls^8^. 

 The use of anticoagulants further elevates the risk of ICH. ICH represents up to 70% of bleeding events associated with oral anticoagulants and is characterized by slow, progressive, and often non-self-limiting bleeding in about half of affected patients^9^. Another risk factor for cerebral hemorrhage is cortical superficial siderosis (cSS). Severe cSS is associated with CAA in leptomeningeal vessels, while milder forms involve cortical vessels. cSS can be evaluated using *ex vivo* and *in vivo* MRI and may serve as a potential neuroimaging biomarker for the diagnosis and prognosis of CAA^10^. In patients with CAA-related ICH, the presence and extent of cSS are strong predictors of recurrence risk (adjusted HR=2.4, 95%CI 1.5–3.7, p<0.0001)^11^. In addition, one study identified a significant association between poor blood pressure control and increased risk of both lobar (HR=3.53, 95%CI 1.65–7.54) and non-lobar (HR=4.23, 95%CI 1.02–17.52) ICH^12^. 

 The definitive diagnosis of CAA is established through histopathological examination, as the disease is characterized by β-amyloid protein deposits in the cerebral vasculature. Prior to the advent of neuroimaging-based diagnostic methods, CAA could only be diagnosed post mortem through brain tissue analysis. Due to the challenges of diagnosing living patients — primarily the inaccessibility of brain tissue — indirect diagnostic approaches became necessary. In the 1990s, the first proposed diagnostic criteria for CAA were introduced in a case report exploring the relationship between CAA and the apolipoprotein E ε4 allele. Greenberg and Charidimou^13^ published a table of diagnostic categories that later became known as the Boston Criteria. These criteria classified CAA into several categories: definitive CAA based on full autopsy findings, *probable CAA with supporting pathology* in clinical contexts with limited brain tissue samples, and *possible and probable CAA (without pathology)* based solely on neuroimaging and clinical exclusion of other causes. 

 These guidelines, now known as the Boston Criteria, were most recently updated in 2022 as the Boston Criteria for Sporadic CAA version 2.0^14^. The prior version, known as the Modified Boston Criteria (2010), introduced a key revision by allowing the inclusion of an additional hemorrhagic lesion beyond lobar regions. In the original version, neuroimaging of hemorrhagic lesions was restricted to lobar brain regions. The modified criteria also included cSS — blood product deposition in cortical sulci — as an additional marker. In the 2022 update, the criteria were expanded to include both hemorrhagic and nonhemorrhagic (white matter) neuroimaging features for the diagnosis of probable and possible CAA, enhancing diagnostic sensitivity and specificity in living patients^14^. 

 The following four diagnostic categories for CAA have been defined: definitive CAA, probable CAA with supporting pathology, probable CAA, and possible CAA. Definitive CAA is diagnosed exclusively through post-mortem histopathological evaluation, confirming the presence of β-amyloid deposition in cortical and leptomeningeal vessels. Probable CAA with supporting pathology involves both clinical and pathological data, with tissue samples obtained from an evacuated hematoma or a cortical biopsy. Clinical manifestations may include spontaneous ICH, transient focal neurological episodes (TFNE), convexity subarachnoid hemorrhage (cSAH), cognitive impairment, and/or dementia. Histopathological analysis must show some degree of CAA and exclude the presence of other lesions that could explain the clinical picture. For probable CAA, only clinical data and neuroimaging are considered. It requires a patient over 50 years of age presenting with spontaneous ICH, TFNE, cognitive impairment, and/or dementia. MRI must show at least two strictly lobar hemorrhagic lesions on T2-weighted sequences (in any combination of: ICH, cerebral microbleeds (CMB), cSS, or cSAH). Alternatively, a single lobar hemorrhagic lesion may be sufficient if accompanied by white matter changes, such as enlarged perivascular spaces in the centrum semiovale or white matter hyperintensities with a multi-pattern distribution. Importantly, no deep (basal ganglia, thalamus, brainstem) hemorrhagic lesions (ICH or CMB) should be present. Additionally, cerebellar hemorrhages are not considered lobar or deep for classification purposes. Possible CAA diagnosis uses the same clinical and radiological criteria as probable CAA but requires only a single lobar hemorrhagic lesion (ICH, CMB, cSS, or cSAH) on T2 sequences or the presence of white matter involvement alone^15^. One study identified that CAA-related ICH has a predilection for posterior brain regions, particularly the occipital and temporal lobes^16^. The patient presented in this case meets the clinical criteria for probable CAA, being over 50 years old and presenting with spontaneous cognitive impairment — specifically short-term amnesia and Broca’s aphasia. In addition, the neuroimaging criteria are met, as MRI (Image A) shows findings consistent with cSS. 

 The prognosis of ICH is generally more favorable when the bleeding is lobar, due to its superficial location and reduced likelihood of intraventricular extension. However, prognosis worsens in cases of extensive bleeding or in aged patients. A cohort study in China found that, after one year of follow-up, 50% of patients had a favorable outcome. Prognosis was influenced by factors such as the initial Glasgow Coma score, ICH recurrence, severe brain atrophy, and a white matter lesion score of 3–4 (indicative of white matter injury)^17^. In this specific case, a notable strength in management was the prompt response of the medical team to complications arising during hospitalization, facilitated by access to comprehensive infrastructure and diagnostic tools. The case was diagnostically challenging, as the patient was oligosymptomatic and initially presented only with mild aphasia. Given the extent of the hemorrhage, the initial working diagnosis was hypertensive ICH. However, cortical hemorrhagic findings on MRI (Image A), which could have suggested CAA, were not initially considered. This omission led to the performance of cerebral angiography — a procedure that might have been avoided had the diagnosis of probable CAA been suspected earlier. This represents a limitation in case management. The differential diagnosis hinges largely on the location of the hemorrhage. In hypertensive ICH, bleeding typically occurs in deep brain structures such as the basal ganglia, brainstem, and cerebellum, whereas in CAA it tends to be lobar and cortical. Although management of both conditions often overlaps — particularly in terms of blood pressure control — prognosis for CAA is worse. CAA carries a higher risk of recurrence, an association with Alzheimer disease, and a lack of disease-specific treatment, making long-term outcomes less favorable compared to hypertensive hemorrhage, for which targeted blood pressure management is more effective. In this case, the patient’s clinical course was favorable. After one year, he demonstrated significant improvement in language and motor function through physical therapy. However, mild cognitive impairment persisted, consistent with long-term outcomes associated with CAA-related ICH. 

 In conclusion, there are various etiologies that can lead to ICH, with the most common differential diagnoses including hypertensive vasculopathy, CAA, and aneurysm rupture. These conditions often present with a range of neurological symptoms such as headache, hemiplegia, loss of consciousness, and other focal neurological signs, which may be progressive and worsen over time. Identifying the underlying cause of ICH is crucial for appropriate management and prognosis. Diagnostic evaluation is typically performed using neuroimaging techniques such as brain CT and MRI. In certain cases, more invasive studies such as cerebral angiography may be necessary to identify causes. These imaging modalities help differentiate the cause of bleeding by identifying specific vascular lesions or by analyzing the location and pattern of the hemorrhage within the brain. 

 In older adults with risk factors — as in the case presented — CAA should be considered as one of the primary differential diagnoses when cerebral bleeding is identified. Early recognition of CAA is essential for optimizing management of the acute event and implementing strategies to control associated risk factors, thereby reducing the likelihood of recurrent hemorrhage. 

 The relevance of this case report lies in highlighting the importance of determining the etiology of ICH, as this directly influences the selection of appropriate diagnostic tests and the development of an effective long-term treatment plan. Accurate diagnosis can significantly improve clinical outcomes and guide follow-up care, especially in patients at high risk of recurrence or cognitive decline. 

## Data Availability

All data sets were generated or analyzed in the current study.
